# Cross-frequency coupling of eye-movement related LFP activities of freely viewing monkeys

**DOI:** 10.1186/1471-2202-12-S1-P132

**Published:** 2011-07-18

**Authors:** Junji Ito, Pedro Maldonado, Sonja Gruen

**Affiliations:** 1 Laboratory For Statistical Neuroscience, RIKEN BSI, 2-1 Hirosawa, Wako, 351-0198 Saitama, Japan; 2 CENI and Programa de Fisiología y Biofísica, ICBM, Facultad de Medicina, Universidad de Chile, Santiago, Chile; 3 Institute of Neuroscience and Medicine, Computational and Systems Neuroscience (INM-6), Research Center Juelich, Juelich, Germany; 4 Theoretical Systems Neurobiology, RWTH Aachen, Aachen, Germany

## 

Living organisms do not only passively receive sensory stimuli from the external world, but also actively explore their surroundings using their sensory organs such as sniffing for odor sensation, whisking for touch sensation, and eye-movements for visual sensation. While neuronal activities underlying active sensing in olfaction and vibrissa sensation have been studied in detail, the activities related to active vision have remained largely unknown. In a recent study, we studied spike synchrony between neurons recorded from the primary visual cortex (V1) of monkeys while they perform visual exploration of natural scene images with self-paced, voluntary eye-movements and found that the V1 cells show excess synchrony around the onset of their response to visual fixation [1]. In the subsequent study, we further examined LFP activities recorded simultaneously with the spike data and found the evidence that the oscillatory LFP activity in the beta frequency band (10-25 Hz) related to the initiation of saccadic eye-movements modulates the timing of single spikes and supports the occurrence of spike synchrony of the V1 cells [2].

It has widely been recognized that brain activities show oscillations on multiple time scales [3]. While previous studies tended to focus on a single, specific frequency band, recent studies have reported strong interactions between activities in different frequency bands [4]. Such interaction is referred to as cross-frequency coupling, and the most common type of coupling is phase-amplitude coupling, also known as nested oscillations [5], where the phase of the slower oscillations modulates the amplitude of the faster oscillations.

In the present study, we investigate cross-frequency coupling of eye-movement related LFPs of freely viewing monkeys. We identify three distinct frequency components characterized by different types of temporal locking to the onset of saccadic eye-movements: A delta-theta band component which is phase-locked to fixation-onset, a beta-gamma band component that is evoked by saccade-onset, and an induced component in the high gamma band. We find that the slowest frequency component (δ–θ (2-8 Hz) frequency band) covaries with the self-initiated, exploratory eye movements. Interactions between this slow component and the other faster components are studied in terms of phase-amplitude coupling measured by the modulation index (MI) [6]. We show that the amplitudes of the faster frequency components (α–β (8-32 Hz) and high-γ (>100 Hz) frequency bands) are modulated by the phase of the slow component (δ band), as reflected in high MI values for the respective frequencies. Furthermore, we find positive correlations between the degree of the phase-amplitude coupling and the amplitude of the slow component.

These results represent the first evidence of mutual entrainment of different LFP frequency components during natural, active viewing behavior. Spontaneous, self-initiated eye-movements are accompanied by a reset of slow oscillations and by evoked beta oscillations, which modulate the high-gamma oscillations induced by visual input. This suggests a structuring role of slow oscillations in the processing of external visual stimuli.
